# Unexpected genomic rearrangements at targeted loci associated with CRISPR/Cas9-mediated knock-in

**DOI:** 10.1038/s41598-019-40181-w

**Published:** 2019-03-05

**Authors:** Amélie Rezza, Christelle Jacquet, Amélie Le Pillouer, Florian Lafarguette, Charlotte Ruptier, Marion Billandon, Patricia Isnard Petit, Séverine Trouttet, Kader Thiam, Alexandre Fraichard, Yacine Chérifi

**Affiliations:** genOway, Lyon, 69007 France

## Abstract

The CRISPR/Cas9 gene editing tool enables accessible and efficient modifications which (re)ignited molecular research in certain species. However, targeted integration of large DNA fragments using CRISPR/Cas9 can still be challenging in numerous models. To systematically compare CRISPR/Cas9’s efficiency to classical homologous recombination (cHR) for insertion of large DNA fragments, we thoroughly performed and analyzed 221 experiments targeting 128 loci in mouse ES cells. Although both technologies proved efficient, CRISPR/Cas9 yielded significantly more positive clones as detected by overlapping PCRs. It also induced unexpected rearrangements around the targeted site, ultimately rendering CRISPR/Cas9 less efficient than cHR for the production of fully validated clones. These data show that CRISPR/Cas9-mediated recombination can induce complex long-range modifications at targeted loci, thus emphasizing the need for thorough characterization of any genetically modified material obtained through CRISPR-mediated gene editing before further functional studies or therapeutic use.

## Introduction

Reverse genetics, the study of phenotypes due to the targeted mutation of a gene, has been and still is largely used to decipher genes or genetic elements function. Historically, this approach was only possible in model organisms where classical Homologous Recombination (cHR) could introduce genomic alterations in Embryonic Stem (ES) cells or zygotes, i.e. mice^1^. Recent advances in gene editing methods with the discovery of ZFNs (Zinc Finger Nucleases), TALENs (Transcription Activator-Like Effector Nucleases), and most recently CRISPR enzymes (Clustered Regularly Interspaced Palindromic Repeats), opened the door to targeted gene modifications in virtually any cell or organism^2^.

CRISPR is a bacterial acquired immune system involving a DNA cleaving enzyme, such as Cas9, guided by a complementary RNA sequence that can specifically recognize and target invaders’ genomic sequences to destroy the invading pathogen^3–5^. This system was adapted to mammalian cells to become a programmable gene editing tool and is now largely and commonly used^6–8^. CRISPR/Cas9, as well as other nucleases, have the property to induce DNA double strand breaks (DSB) at targeted sequences, resulting in a cellular response to maintain genome integrity. Two repair mechanisms prevail: Non-Homologous End Joining (NHEJ) which produces small insertions or deletions (indels) at the cleavage site, or Homologous Directed Repair that induces the specific insertion of an exogenous DNA fragment at the cut site. Hence, CRISPR/Cas9 has been shown to be efficient for Knock-Out (KO) and Knock-In (KI) in cells and zygotes of different species^9–13^. It does however show some limitations. Different groups have observed its limited robustness and reproducibility for insertions of large inserts (>1.5 kb), and it has been consistently observed that the CRISPR/Cas9 system in zygotes produces mosaic founders^14,15^, involving further breeding to obtain heterozygous and homozygous animals.

Here we performed more than 220 experiments to insert DNA fragments >1.5 kb in more than 120 loci, distributed along the 19 autosomes and X chromosome of mouse ES cells, using either CRISPR/Cas9 or cHR (Sup Table). We systematically used Streptococcus pyogenes (Sp) Cas9 nickase (Cas9n) for all experiments to specifically target the intended insertion site while limiting off-target activity^16–18^. While recombined clones could be obtained with both technologies, we confirmed that Cas9n reproducibly gives more positive clones than cHR as detected by overlapping PCRs. However, cHR allowed the identification of a significantly higher ratio of fully targeted clones, as validated by Southern blot (SB) and short-range sequencing. This greater efficiency of cHR over Cas9n was observed in all tested conditions, except for inserts of more than 8 kb where both technologies proved equivalent. These observations were made not only for the targeting of the *Rosa26* locus, but were also validated over all tested loci, suggesting that CRISPR/Cas9-mediated KI in mouse ES cells is more efficient than cHR for the detection of recombined clones, but that cHR is more robust and efficient for error-free insertions. As a preliminary explanation for this observation, we looked at SBs performed on PCR positive clones from Cas9n or cHR experiments at different loci. For Cas9n-mediated KI, we observed a significant increase in the number of experiments and clones showing bands of unexpected sizes when using an external probe, i.e. insertion at the targeted locus. This observation suggests the presence of undesired rearrangements at the targeted loci when using CRISPR/Cas9 technology, rearrangements that never occur when using cHR. Interestingly, a recent study reported similar observations showing that Cas9-mediated DSB alone induced complex rearrangements in two targeted loci^19^. We report here that a similar phenomenon may also occur with Cas9 nickase and in the presence of donor DNA, in more than 40% of tested loci (25 out of 56).

Taken together these data show that both technologies can efficiently yield recombined clones. However, CRISPR/Cas9 technology increases DNA insertion efficiency at the targeted locus (% overlapping PCR^+^ clones), but also yields a lower frequency of fully validated recombined clones (% SB^+^ and Seq^+^ clones). Particularly, the recurring presence of additional bands on SB performed with external probes when using Cas9n suggests that genomic rearrangements have occurred at the targeted locus. This study show that all materials obtained with CRISPR/Cas9 technology must be carefully characterized by SB and/or long-range sequencing at the targeted locus before further use.

## Results

### Efficiency comparison of Cas9n and classical HR in mouse ES cells

Mouse ES cells are highly recombinant cells that allow efficient targeted integration of exogenous DNA by cHR. In an effort to optimize KI efficiency in these cells, we systematically compared cHR and CRISPR/Cas9 technology. In order to lower off-target cuts and indels, we exclusively used paired Sp Cas9 nickases D10A, Cas9n, to introduce DSBs at the targeted locus.

First focusing on the extensively used and studied *Rosa26* locus, we introduced inserts ranging from 3 to 12 kb into its first intron using Cas9n or cHR (Cas9n n = 22; HR n = 6). A unique guide-RNAs pair was used for all Cas9n experiments. Recombined clones were first screened by overlapping 5′PCR, then confirmed with overlapping 3′PCR. Southern blot (SB) and short-range sequencing were used to definitively identify properly targeted clones. As previously shown, the percentage of positive clones detected by 5′PCR was superior when using Cas9n to that obtained with cHR (Fig. S1A), whereas cHR’s percentage of clones validated by short-range sequencing was superior to Cas9n’s (Fig. S1D). Although no significant difference was observed, the percentage of positive clones detected by 3′PCR appeared higher when using Cas9n than cHR (Fig. S1B), while cHR’s percentage of clones validated by SB seemed superior to Cas9n’s (Fig. S1C).

**Figure 1:**
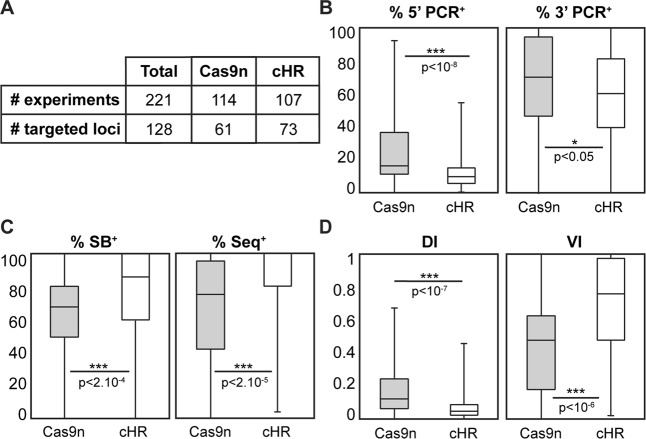
Comparison of cHR and Cas9n knock-in performance in mouse embryonic stem cells. (**A**) Number of experiments and loci targeted in mouse ES cells using Cas9n technology (Cas9n) and cHR (see Sup Table for further details). (**B**) Box plots of percentages of positive clones detected by 5′PCR (left) or 3′PCR (right) for all experiments at all loci using Cas9n or cHR. (**C**) Box plots of percentages of clones validated by Southern Blot (SB; left) or sequencing (right) of the targeted locus for all experiments at all loci using Cas9n or cHR. (**D**) Box pots of Discovery Index (DI, left) and Validation Index (VI, right) for all experiments at all loci using Cas9n or cHR. DI and VI represents the technology’s efficiency to produce PCR-positive clones and fully validated clones, respectively.

To investigate further these observations, we compared KI experiments in 128 different loci distributed along the 20 murine chromosomes, using either Cas9n or cHR (Fig. 1A and Sup Table). As observed at the *Rosa26* locus, the percentage of clones detected by overlapping PCRs was higher with Cas9n than with cHR (Fig. 1B), whereas the percentage of fully targeted clones was higher with cHR than with Cas9n (Fig. 1C). These data confirm that the use of Cas9n significantly increases the number of PCR detected clones, but yields lower percentage of fully recombined clones than cHR, as validated by SB and short-range sequencing.

To better compare both methods, we defined efficiency indexes to represent their performance. The Discovery Index or DI, illustrating the potential of the technology to yield recombined clones as per PCR detection was significantly higher with Cas9n than cHR (Fig. 1D).The Validation Index or VI, representing the technology’s ability to give fully validated recombined clones was significantly higher with cHR than Cas9n (Fig. 1D), showing that although fewer clones are initially detected with cHR, those are more likely to be fully validated than the clones detected using Cas9n. These trends were also observed, although not significant, at the *Rosa26* locus (Fig. S1E).

All together, these data show that Cas9n technology does give more clones to screen than cHR, but these clones are less likely to contain the expected recombination event devoid of any other genomic alteration or mutation.

### Insert size impact on Knock-In efficiency using Cas9n or classical HR in mouse ES cells

**Figure 2:**
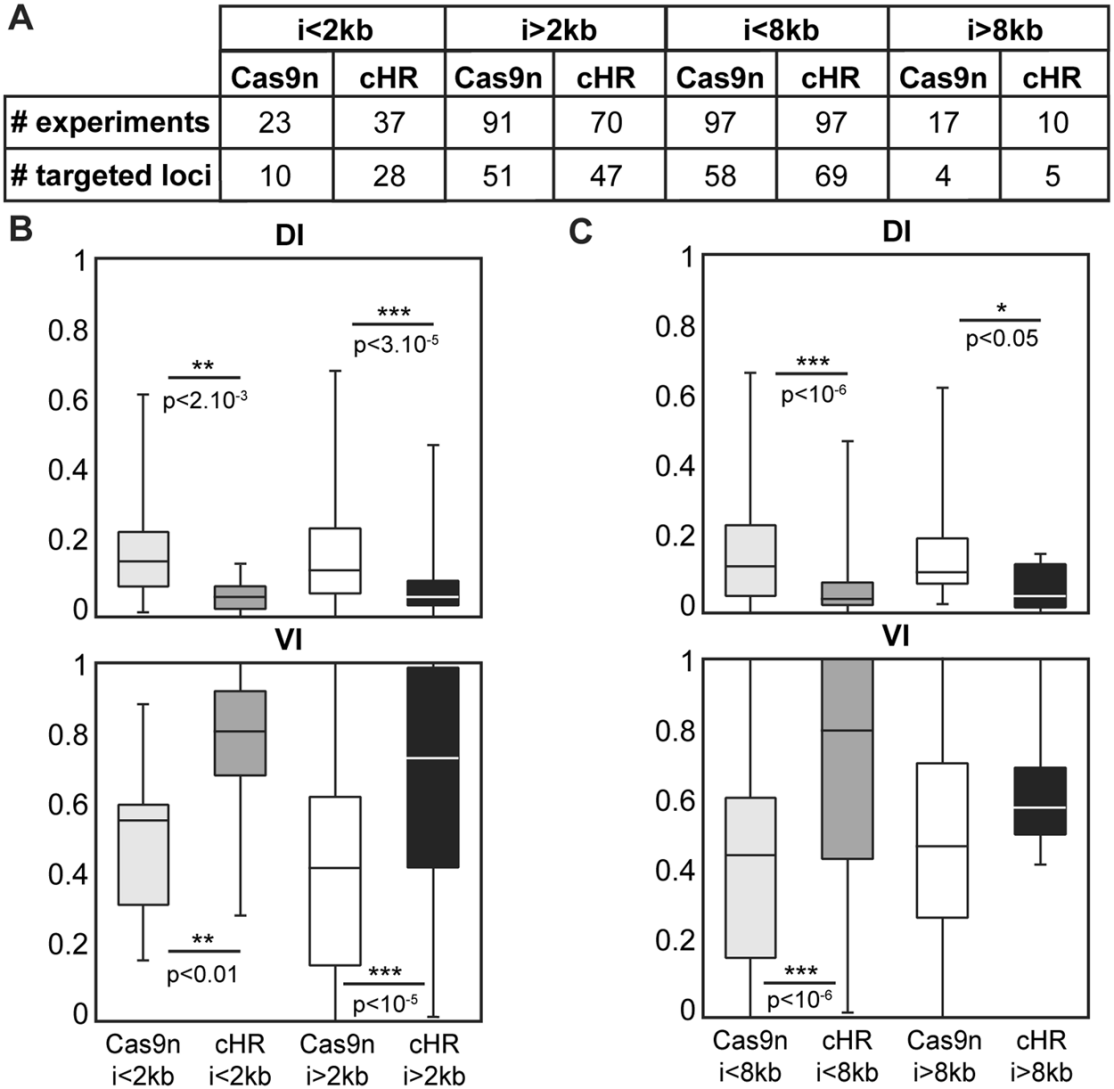
Knock-In efficiency for inserts of different size with Cas9n and cHR. (**A**) Number of experiments and loci targeted in mouse ES cells using Cas9n technology or cHR for inserts of various size (see Sup Table for further details). (**B**) Box plots of Discovery Index (DI, top) and Validation Index (VI, bottom) for small (<2 kb) and bigger inserts (>2 kb). (**C**) Box plots of Discovery Index (DI, top) and Validation Index (VI, bottom) for large (>8 kb) and smaller inserts (<8 kb). Note that Cas9n’s DI is superior to cHR’s for all insert sizes whereas cHR’s VI is higher than Cas9n’s in all conditions except for inserts >8 kb.

Insert size is commonly known to be a major limiting factor to efficient homologous recombination, at least at some loci. We aim here to definitively illustrate this observation by comparing Cas9n and cHR efficiency in recombining inserts of various sizes at different loci (Fig. 2A).

First focusing on small inserts (<2 kb), we found again that Cas9n gave significantly more PCR detected clones than cHR (Figs 2B top panel and S2A,B), but fewer fully validated clones (Figs 2B bottom panel and S2C,D). The same observations were made for inserts 2 kb (Figs 2B and S2A–D). Although both indexes were similar for both insert sizes (Fig. 2B, Cas9n <2 kb vs >2 kb), Cas9n %Seq^+^ clones was significantly higher for small inserts (Fig. S2D), suggesting that Cas9n is most efficient for inserts <2 kb.

Similar results were observed for inserts <8 kb (Figs 2A,C and S2E–H). Interestingly for large inserts (>8 kb), we did not observe a significant difference between Cas9n’s and cHR’s VI (Fig. 2C), suggesting that both technologies have similar KI efficiency for inserts >8 kb. This confirms previous observations that cHR could be less efficient for large inserts than smaller ones (Fig. 2C: HR <8kb vs HR >8 kb), although no significant difference was observed.

Taken together, these data confirmed our previous observations that Cas9n allows for the initial detection of more clones while cHR shows better efficiency for the generation of fully validated clones, except for large inserts (>8 kb).

### Impact of homology arm length and nuclease concentration on Cas9n Knock-In efficiency

Cas9n technology’s efficiency may be affected by many parameters including homology arm length and nuclease concentration. We adjusted these parameters in an attempt to improve Cas9n performance in mouse ES cells.

**Figure 3:**
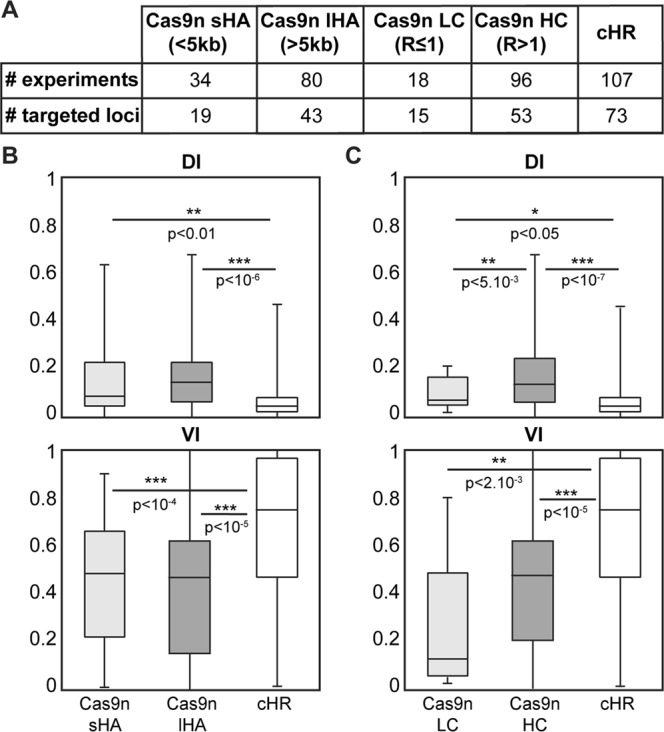
Homology arm length and Cas9n enzyme concentration effect on Cas9n efficiency. (**A**) Number of experiments and loci targeted in mouse ES cells using Cas9n technology with short (sHA) or long homology arms (lHA), low (LC) and high concentration (HC) of enzyme, and cHR (see Sup Table for further details). (**B**) Box plots of Discovery Index (DI, top) and Validation Index (VI, bottom) for sHA and lHA. (**C**) Box plots of Discovery Index (DI, left) and Validation Index (VI, right) for LC and HC. Note that HA length does not affect Cas9n’s efficiency. HC shows higher DI than LC but the VI is still inferior to cHR’s. R: μg Cas9n/million cells.

We first tested different lengths of homology arms (HA, Fig. 3A). The efficiency of Cas9n when using short HAs (sHA: <5 kb total) was compared to Cas9n’s efficiency when using long HAs (lHA: >5 kb total). As previously observed, both Cas9n conditions proved more efficient than cHR for the production of PCR-positive clones, whereas cHR yielded more fully validated clones than either Cas9n condition (Figs 3B and S3A). Additionally, no significant difference was observed between sHA and lHA, suggesting that Cas9n’s performance does not depend on HA length. Taken together, these results show that the length of the homology arms used for recombination when considering different loci all together does not significantly influence Cas9n efficiency. Importantly, cHR showed a higher VI compared to any group of HA length used with Cas9n, here again confirming the superiority of cHR over Cas9n for final validation of recombined clones.

We also tested the effect of nuclease concentration on KI efficiency. Different concentrations, ranging from 0.5 to 2.7 μg/million cells of Cas9n expressing plasmid were tested to target different loci. Efficiency indexes were compared for all tested loci. Data showed that both Cas9n low and high concentrations (LC and HC respectively) again gave higher DI than cHR, whereas cHR showed the highest VI (Fig. 3C), confirming its superiority over Cas9n for definitive clone validation. Interestingly, Cas9n LC was significantly less efficient than HC for clone detection, as illustrated by lower %5′PCR^+^ clones (Fig. S3B) and DI (Fig. 3C), and showed a lower %SB^+^ clones than HC (Fig. S3B). This suggests that low concentrations of Cas9n expressing plasmids are not efficient for the production of targeted KI in mouse ES cells. No significant difference in efficiency was observed between medium (between 1 and 2) and highest concentrations (≥2), showing that very high concentrations of Cas9n do not increase KI efficiency (Fig. S3C,D).

Thus, even the highest concentrations of Cas9n did not out-perform cHR for clone validation efficiency, confirming previous observations. Importantly, high concentrations of Cas9n may give better results for clone detection than lower ones, but they also likely favor off-targets as high concentrations of Cas9 WT have been shown to increase off-targets^20,21^.

### Cas9n induces unexpected rearrangements at targeted loci in mouse ES cells

In order to understand why many PCR positive clones were later invalidated when using Cas9n, we looked at SB validation using loci specific probes. Using an «external probe» specific to the targeted locus and hybridizing right outside the homology arms, we could detect the correct insertion of the donor DNA through the presence of a specific band at an expected size (Recombined allele: Rec).

Looking first at a single locus, namely *Rosa26*, we performed SB with specific external probes for 11 Cas9n experiments and 6 cHR experiments (Fig. S4A). All blots for cHR experiments showed only WT and Rec expected bands (Fig. S4B). Six blots for Cas9n experiments also showed exclusively WT and Rec bands for all clones (not shown), whereas 5 Cas9n experiments’ blots displayed bands of unexpected sizes (Fig. S4C, clones b and d). Overall, the average percentage of clones showing these unexpected bands for Cas9n experiments at the Rosa26 locus was 9% (Fig. S4D), significantly more than for cHR experiments which did not show any.

**Figure 4:**
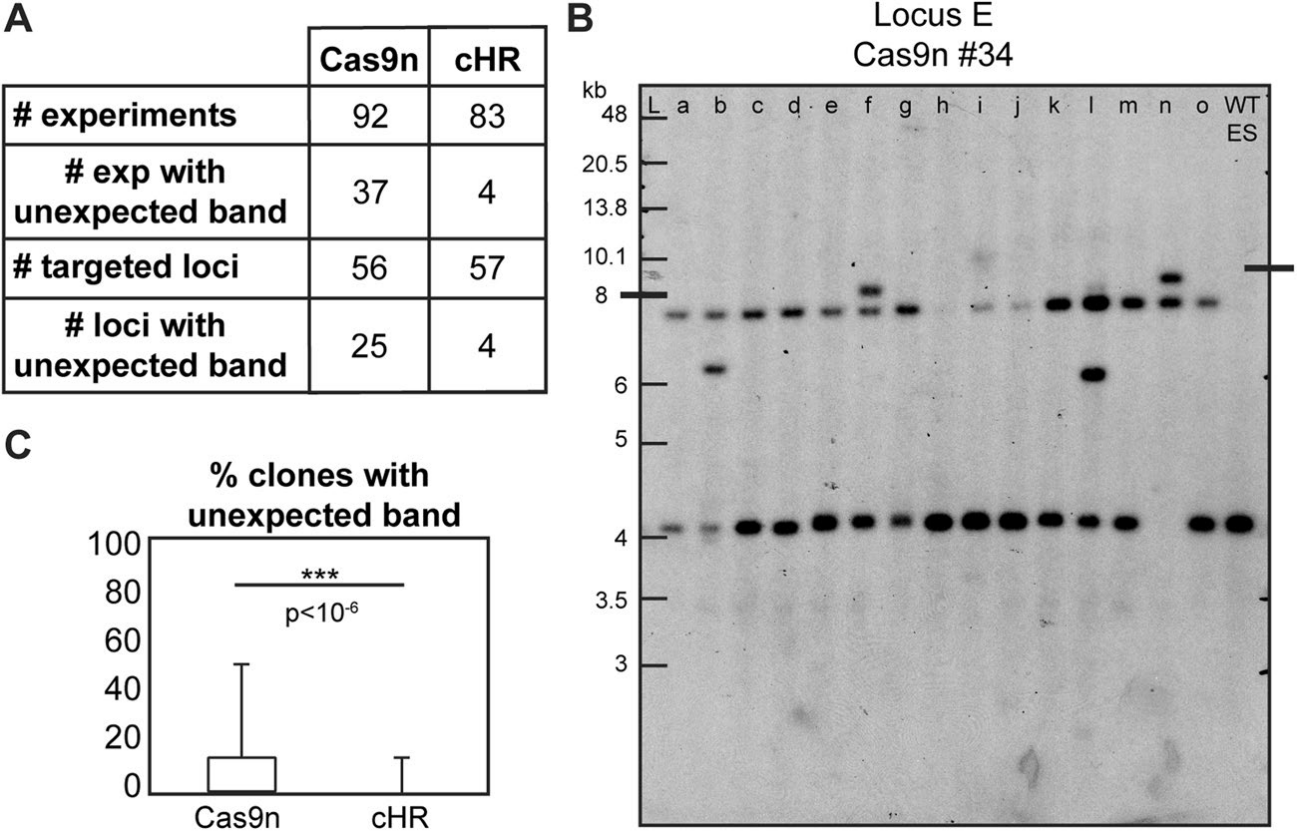
Cas9n technology induces unexpected rearrangements at the targeted locus. (**A**) Number of experiments and loci targeted in mouse ES cells using Cas9n technology and cHR, tested by Southern Blot (SB) with an external probe (see Sup Table for further details). (**B**) Blot of Cas9n experiment #34 targeting locus E showing profiles obtained with an external probe after EcoNI digestion (WT: 4011; Rec: 7108). Note that clones 34b, 34 f, 34i, 34 l and 34n show bands of unexpected size. (**C**) Box plots of percentages of clones showing bands of unexpected sizes by SB with Cas9n and cHR for all loci. L: ladder.

To validate this observation at other loci, we performed external SBs for 175 experiments using Cas9n or cHR in a total of 109 different loci (Fig. 4A and Sup Table). Here again, blots for cHR experiments showed WT and Rec expected bands (Fig. S4E), and very rarely unexpected displaced signals (4/83 = 4.8%; Fig. S4F clone b). In contrast, experiments performed with Cas9n showed additional bands of unexpected sizes on 37 blots out of 92 (40.2%; Figs 4B and S4G) whereas all other blots only showed WT and Rec bands (Fig. S4H). Moreover, the average percentages of clones showing unexpected bands were 0.5% for cHR and 7.6% for Cas9n projects (Fig. 4C), a significant 15-fold increase.

The presence of bands of unexpected sizes, particularly additional bands, on blots performed with external probes for clones detected positive by overlapping PCRs suggests some rearrangement at the targeted locus. Thus, these data suggest that Cas9n-mediated recombination can induce unexpected rearrangements at the targeted locus in mouse ES cells. This is in accordance with a recently published study showing that repair of Cas9-mediated DSB induces complex rearrangements at the targeted locus^19^.

## Discussion

The discovery of the CRISPR/Cas9 system and its adaptation into a performing genome modifying tool has revolutionized gene editing but also opened very promising avenues for regenerative medicine and gene therapy. Since its inception, a great deal of attention has focused on the risk of off-targets inherent to CRISPR/Cas9 technology, and many optimizations are being established to minimize this risk and make this technology safe for medical applications^22^. Interestingly, less focus has been centered on the thorough characterization of its effect on targeted loci, especially on a large scale. CRISPR/Cas9’s efficiency and superiority to other methods has been extensively studied in numerous works, but it is most often studied in a limited number of loci. Recently, a large-scale systematic approach on 81 genome editing projects was used to study off-targets for the generation of KO in mouse and rat zygotes^23^, but the present study is, to our knowledge, the first to highlight “on-target” CRISPR-mediated modifications on such a large scale.

To try and compare Cas9n and cHR efficiency in mouse ES cells, rather than evaluating their performance in parallel assays at the same locus (which would call for several repeats for each locus to have significant results, and would only apply to this specific locus), we chose to look at experiments performed at different loci all together. Here we found that using paired Cas9n for insertion of large fragments at first appears more efficient than cHR as detected by overlapping specific PCRs. However, when thoroughly characterizing the clones obtained with both technologies, we found that cHR robustly and reproducibly resulted in more correctly recombined clones as validated by Southern blot (SB) and short-range sequencing. In other words, Cas9n yields more PCR^+^ clones to screen, but a lower percentage of those actually show the correct insertion at the targeted locus. To our knowledge, this observation has never been made so far, most likely because few studies have compared side by side CRISPR/Cas9 system and classical HR for large DNA insertions, in such a vast number of loci and experiments and with such a systematic approach (PCR screening, SB and short-range sequencing characterization). We also looked at different parameters to try and identify if these observations could be restricted to specific conditions. We showed that neither HA length, nor Cas9n concentration had a positive effect on the technology’s efficiency to produce fully validated clones, as cHR remained superior to all Cas9n conditions tested. We did observe some restrictions depending on insert size, as some trends were observed, although not significant: (1) Cas9n is more efficient for small inserts (<2 kb) than for larger ones, albeit less efficient than cHR for both insert sizes; (2) cHR is less efficient for large inserts (>8 kb) than for smaller ones, while still equivalent to Cas9n. A recent study in zygotes showed promising results for targeted KI using long single stranded DNA donors but it proved to be limited to the insertion of fragments up to 2 kb only^24^. It is also important to note that extensive sequencing to validate recombined embryos was only performed on a small number of individuals in this prior study, and additional in-depth characterization of material obtained with this technique is required to definitively conclude on its efficiency.

Our systematic characterization of recombined clones by SB showed that experiments performed with Cas9n displayed unexpected bands when analyzed with a locus-specific external probe. Indeed 44% of Cas9n-targeted loci showed unexpected bands (25/56 tested loci; Sup Table), whereas only 7% of loci targeted with cHR displayed such bands (4/52 tested loci). Moreover, the unexpected bands observed for clones from cHR were in fact shifted bands (Fig. S4F clone b) and not additional bands, suggesting the insertion of truncated or partially duplicated donor fragments. On the contrary, bands observed on blots of Cas9n experiments (88 clones from 37 blots,) were most often additional bands (Figs 4B clones b, f and l, S4C clone d and S4G clones e, I, n, p, q and s). The specific rearrangements occurring at targeted loci still need to be identified by long-range sequencing but the SB results shown here suggest the presence of complex rearrangements that could be similar to the one observed in Kosicki *et al*.^19^ (combinations of multiple insertions, deletions, translocations and/or inversions). Interestingly, such additional bands were observed for experiments performed with a wide range of Cas9n concentration (from 0.5 to 2.7 μg/million cells), suggesting the molecular mechanism responsible for their occurrence, although still unknown, is not affected by nuclease concentration. Additionally, with our protocol CRISPR/Cas9 produces heterozygous ES clones (presence of WT and Rec bands on blots). We can then hypothesize that the non-recombined allele might also be carrying indels after Cas9n cuts due to NHEJ-repair, whereas it most likely remains unaltered when using cHR, as no DNA cut occurred.

Only one prior recent study reported the occurrence of complex rearrangements at the DSB after Cas9 cut^19^. This phenomenon was observed in mouse ES cells, a human cell line, and primary mouse hematopoietic progenitors, when using Cas9 plasmid or ribonucleoprotein complex. No donor DNA was used in the study and, importantly, only 3 loci were analyzed in the different models. Here, we show that long-range genomic modifications, potentially similar to these rearrangements, can also occur when DNA is integrated at the DSB, with Cas9n, at 25 out of 56 loci analyzed.

The observation that CRISPR/Cas9 technology induces complex rearrangements at targeted loci has also been reported in studies aiming at producing large deletions or inversions in mouse zygotes and ES cells^25,26^. By using two Cas9 targeting specific distant sites and thus giving two DSBs, both groups have shown that uncontrolled rearrangements can occur rendering the characterization of the specific genetic modifications more complex. Our data suggest that similar unwanted rearrangements may also occur following a single DSB with cohesive ends.

In conclusion, we showed that although both technologies can efficiently produce KI clones, CRISPR/Cas9 technology is clearly more efficient than cHR for the insertion of large DNA fragments as detected by PCR screening. We also showed that Cas9 technology can induce yet still uncharacterized, unwanted genomic rearrangements at the targeted locus, eventually rendering Cas9n less efficient than cHR for the production of fully validated recombined clones. Further investigations are still needed to fully characterize the rearrangements responsible for the observed additional bands on SB, to eventually better understand the molecular mechanism involved in their occurrence. However, this work underlines the necessity to carefully design experimental conditions and thoroughly characterize any material obtained through CRISPR/Cas9-mediated modifications, for potential off-targets, but also at the targeted locus. Systematic and rigorous characterization of Cas9-mediated obtained materials will increase our understanding of CRISPR/Cas9 technology, and help make it an efficient, safe gene editing tool especially for both basic science and therapeutic applications.

## Methods

### Plasmids

Guide-RNAs design tool was develop in house. Paired guide-RNAs were designed to induce nicks separated by a maximum of 20 nucleotides. A minimum of one guide-RNA cut site was destroyed by insertion of the donor DNA (exogenous DNA part of the insert). Guide-RNAs with highest score were chosen and inserted in BpiI linearized pX335 vector.

Donor vectors contained various homology arms sizes of isogenic DNA (Sup Table), and isogenic and/or exogenous DNA in the insert fragment. All donor vectors contained a Neomycin resistance cassette in the insert, as well as a Diphteria Toxin A expression cassette in the vector backbone. Total homology arm’s length is indicated for each experiment in Sup Table. Distribution of the homology arms varied from 1:1 to 1:3 for both Cas9n and cHR experiments. Homology arms and DNA fragment inserts were cloned in pBluescript II KS for Rosa experiments and pSmart HC for all other loci.

### Cell culture and electroporation

C57Bl6/N male mouse ES cells were obtained from Thromb-X (Belgium), cultured on mitomycin treated-mouse embryonic fibroblasts in adapted 3i medium^27^ at 37 °C and 7% CO_2,_ and passaged at subconfluence. ES cells at approximately 80% confluence were transfected using BTX ECM 630 electroporator in 4 mm cuvette. For Streptococcus pyogenes Cas9n experiments, cells were electroporated with 3 plasmids: 2 coding for Cas9n protein and a specific guide RNA, and the linear targeting vector carrying the DNA to be inserted. We defined low Cas9n concentration (LC) as inferior or equal to 1 μg of Cas9n expressing plasmid per million cells, and high concentration (HC) as superior to 1. For cHR experiments, cells were electroporated with the linearized donor DNA plasmid (ratio ug plasmid/million cells from 1 to 3). Cells were then cultured in medium plus Neomycin for 2 weeks to obtain clones. Clones were picked and amplified for further genotyping and storage. All experiments were performed in accordance with the Direction générale de la recherche et de l’innovation regulations with approval from the Ministère de l’éducation nationale, de l’enseignement et de la recherche ethical committee (approval #A212230105).

### PCR genotyping

For each locus and experiment, 5′-PCR primers were designed to amplify an overlapping region at the 5′ end of the insert: forward primer hybridizing outside the 5′ homology arm, reverse primer hybridizing inside the insert. Similarly, 3′-PCR primers amplified an overlapping region at the 3′ end of the insert (forward primer hybridizing in the insert, reverse primer hybridizing outside the 3′ homology arm). All PCR primers were designed using proprietary software. PCR fragments sizes were checked using ZAG DNA Analyzer capillary electrophoresis (AATI).

### Southern blot hybridization

Southern blot was performed using purified genomic DNA using specific internal probes (for validation of targeted insertions and detection of potential ectopic insertions) and external probes (for validation of correct full insertions at the targeted locus), as previously described^28^.

### Sequencing

Junctional DNA fragments overlapping Cas9n original cut sites on both sides of the insertion, as well as entire transgenes (when present) were sequenced. PCR products were sent to Biofidal, Lyon for Sanger DNA sequencing. Sequencing runs with overlapping sequence were aligned using VNTI (Thermo Fisher scientific) software. Sequenced ES cell clones showing integration of the entire transgene together with indels or mutations within junctional DNA fragments were not considered positive.

### Efficiency indexes calculation

Efficiency indexes were used to best represent Cas9n and cHR technology performance. The discovery index (DI), representing the efficiency of the technique to generate PCR positive clones i.e. a «1st screen» recombination efficiency was calculated as %5′-PCR^+^/100 × %3′-PCR^+^/100.The validation index (VI), illustrating the efficiency of the technique to produce fully validated clones, was calculated as %SB^+^/100 × %Seq^+^/100.

### Statistics

The exact percentages of positive clones for each individual experiment are reported in Sup Table, as well as the n for all tested conditions. Statistical analysis was performed using the GraphPad Prism 6.0 and Microsoft Excel computer programs. The results are represented as box plots of percentages and ratio. The ends of the box are the upper and lower quartiles, so the box spans the interquartile range (IQR). The median is marked by a vertical line inside the box. The ends of the whisker are set at 1.5*IQR above the third quartile (Q3) and 1.5*IQR below the first quartile (Q1). Unpaired two-tailed Student’s *t*-test were used to assess differences between two groups, and a value of *P* < 0.05 was considered statistically significant.

## Supplementary information

Supplementary information

Supplementary Table

## Data Availability

Targeted loci were selected amongst previous customers' loci of interest and were thus anonymized. All relevant data are shown in figures, Sup table and available from the authors upon request.
