# Supplementary material for: Unexpected genomic rearrangements at targeted loci associated with CRISPR/Cas9-mediated knock-in
Source: Sci Rep. 2019 Mar 5;9:3486. doi: 10.1038/s41598-019-40181-w (PMC6401152; doi:10.1038/s41598-019-40181-w)

Supplementary information

Unexpected genomic rearrangements at targeted loci associated with CRISPR/Cas9-mediated knock-in

Amélie Rezza, Christelle Jacquet, Amélie Le Pillouer, Florian Lafarguette, Charlotte Ruptier, Marion Billandon, Patricia Isnard Petit, Séverine Trouttet, Kader Thiam, Alexandre Fraichard, Yacine Chérifi

Affiliation
genOway, Lyon 69007, France

List of supplementary items

Figure S1 related to Figure 1
Figure S2 related to Figure 2
Figure S3 related to Figure 3
Figure S4 related to Figure 4
Supplementary Table (Excel file)

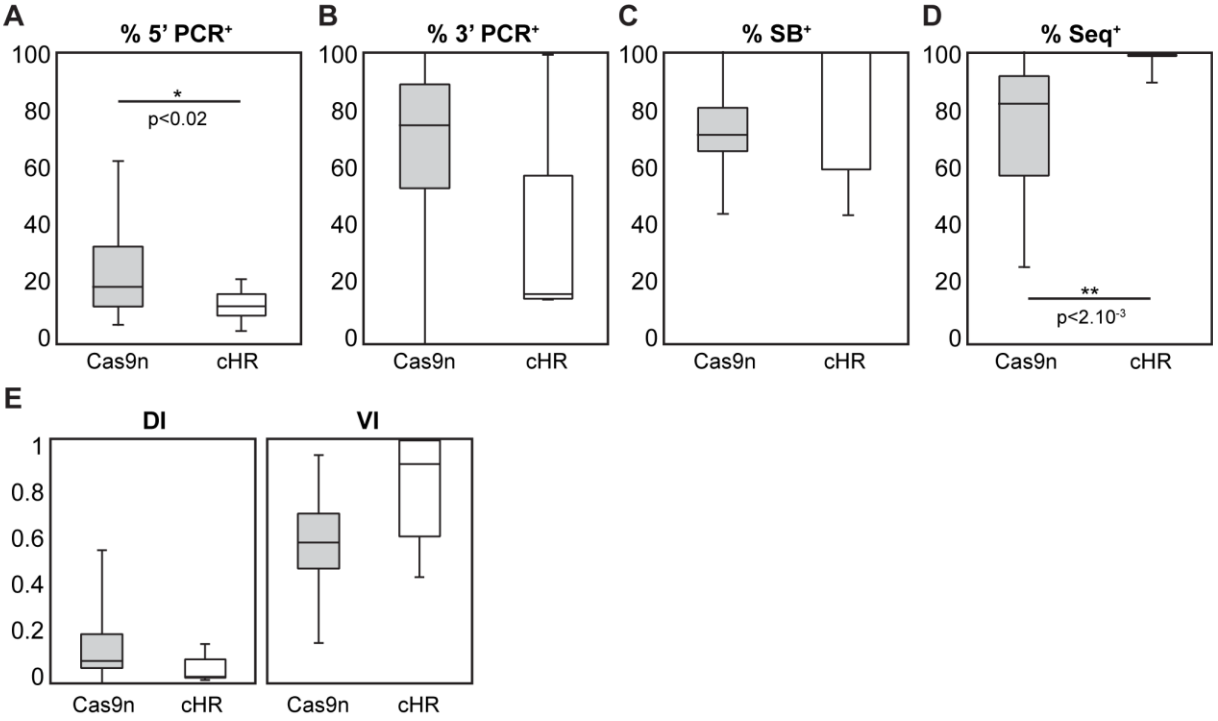

Figure S1: Comparison of cHR and Cas9n knock-in performance at the Rosa26 locus, in mouse ES cells. Box plot representation of the percentages of positive clones detected by 5'PCR (**A**), 3'PCR (**B**), validated by Southern Blot (**C**) or sequencing (**D**) for all experiments using Cas9n (n=22) or cHR (n=6). **E.** Box plots of Discovery Index (DI, left) and Validation Index (VI, right) for all experiments in the Rosa26 locus using Cas9n or cHR. DI and VI represent efficiency to produce PCR-positive clones and fully validated clones, respectively.

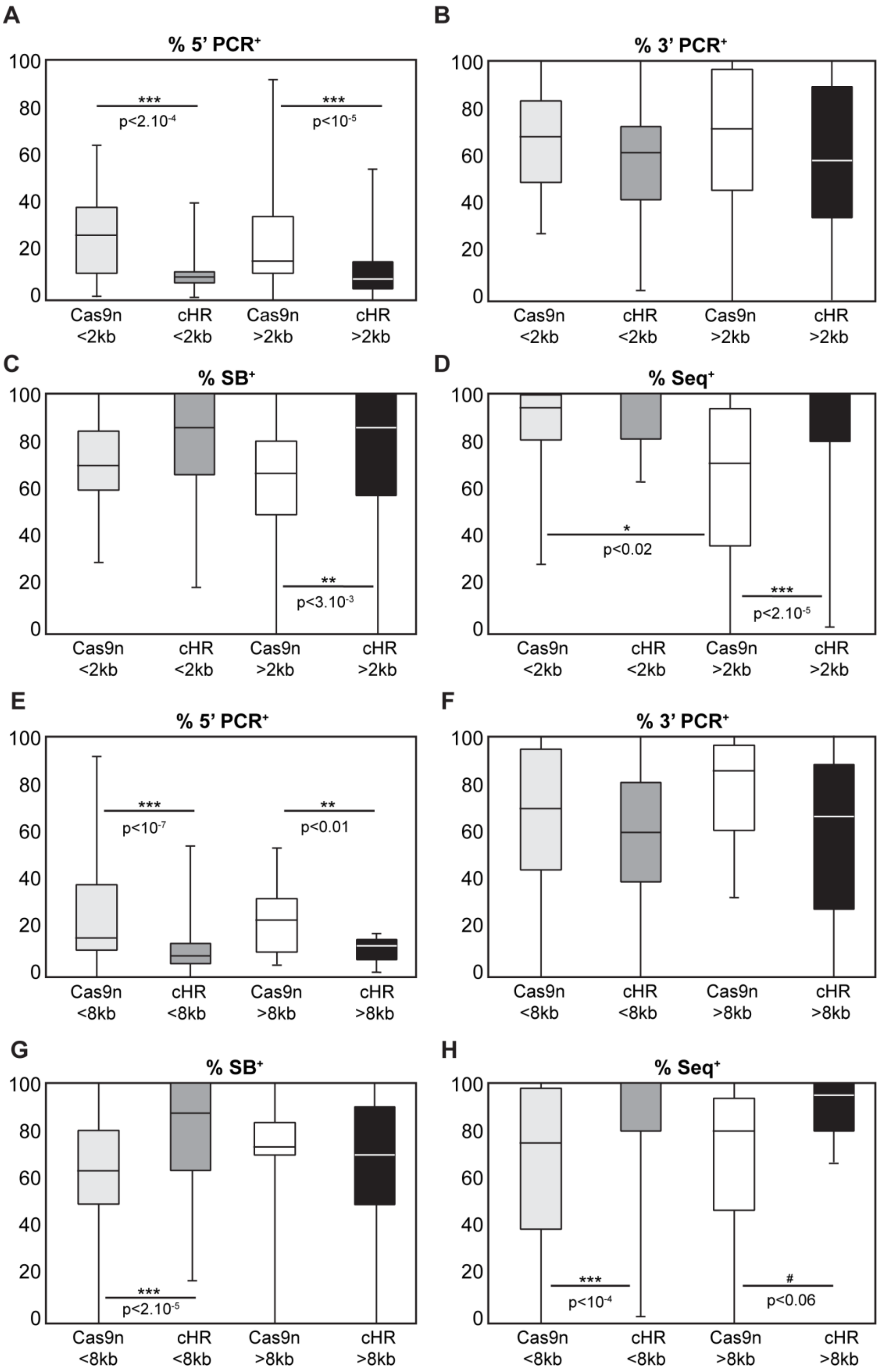

Figure S2: Effect of insert size on percentage of detected and validated clones using Cas9n or cHR. Box plot representation of the percentages of positive clones detected by 5'PCR (**A**), 3'PCR (**B**), validated by Southern Blot (**C**) or sequencing (**D**) for small (<2kb) or larger inserts (>2kb) with Cas9n or cHR. Box plot representation of the percentages of positive clones detected by 5'PCR (**E**), 3'PCR (**F**), validated by Southern Blot (**G**) or sequencing (**H**) for large (>8kb) or smaller inserts (<8kb) with Cas9n or cHR.

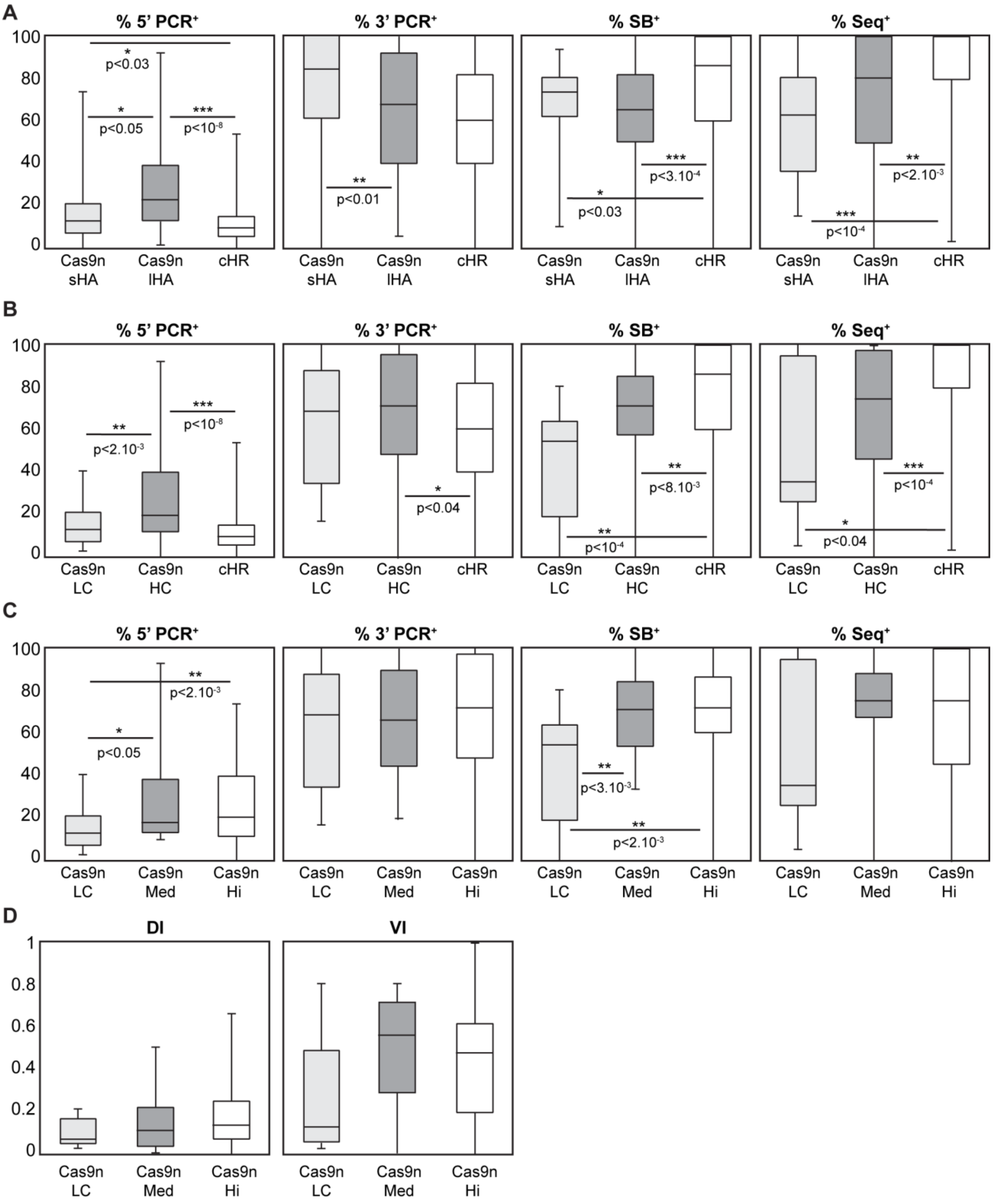

Figure S3: Optimization of Cas9n performance through homology arm length and Cas9n concentration.

A. Box plot representation of the percentages of positive clones detected by 5'PCR, 3'PCR, validated by SB and sequencing for all experiments at all loci with Cas9n sHA, Cas9n lHA or cHR. **B.** Box plot representation of the percentages of positive clones detected by 5'PCR, 3'PCR, validated by SB and sequencing for all experiments at all loci with low concentration (LC; R≤1) of Cas9n, high concentration (HC; R>1) of Cas9n or cHR. **C.** Box plot representation of the percentages of positive clones detected by 5'PCR, 3'PCR, validated by SB and sequencing for all experiments at all loci with medium concentration (Med; 1<R<2; n=16) of Cas9n, highest concentration (Hi; R≤2; n=80) of Cas9n or LC. **D.** Box plots of Discovery Index (DI, left) and Validation Index (VI, right) for LC, Med and Hi. R: µg Cas9n plasmid/million cells.

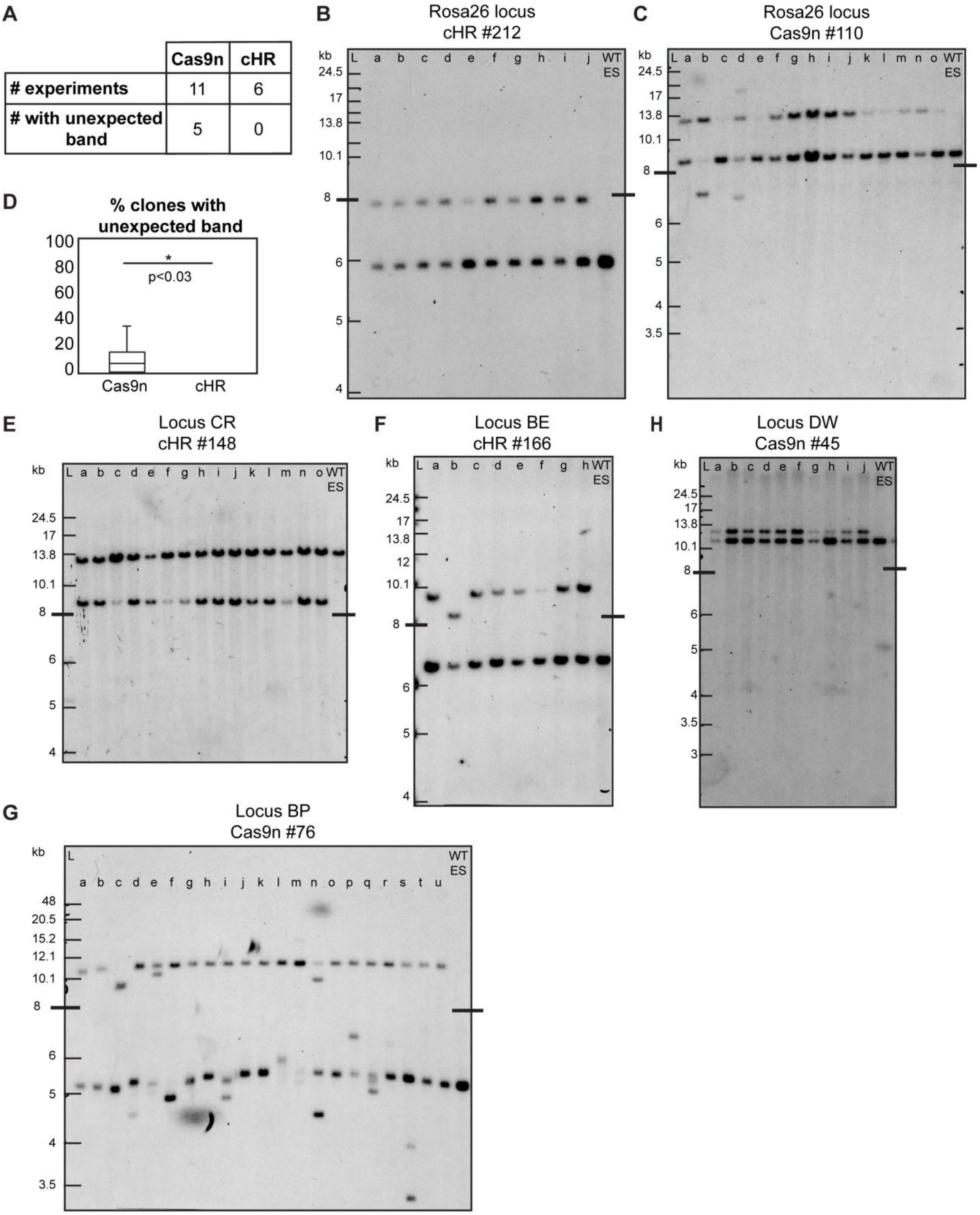

Figure S4: Thorough characterization of clones obtained with cHR and Cas9n by southern blot. A. Number of experiments targeting the Rosa26 locus in mouse ES cells using Cas9n technology or cHR, tested by Southern Blot (SB) with an external probe. **B.** Blot of cHR experiment #212 targeting Rosa26 locus showing profiles obtained with an external probe after BamHI digestion (WT: 5825bp; Rec: 7620bp). **C.** Blot of Cas9n experiment #110 targeting Rosa26 locus showing profiles obtained with an external probe after AspI and NheI

digestion (WT: 8487bp; Rec: 12332bp). Note that clones 110b and 110d show bands of unexpected size. **D.** Box plot representation of the percentages of clones showing bands of unexpected sizes by SB using Cas9n or cHR to target the Rosa26 locus. **E.** Blot of cHR experiment #148 targeting locus CR showing profiles obtained with an external probe after AflII digestion (WT: 12893bp; Rec: 8671bp). **F.** Blot of cHR experiment #166 targeting locus BE showing profiles obtained with an external probe after BamHI digestion (WT: 6466bp; Rec: 9219bp). Note that clone 166b shows a band of unexpected size. **G.** Blot of Cas9n experiment #76 targeting locus BP showing profiles obtained with an external probe after BamHI digestion (WT: 5281; Rec:11083). Note that clones 76a, 76c, 76d, 76e, 76f, 76i, 76l, 76n, 76p, 76q and 76s show bands of unexpected size. **H.** Blot of Cas9n experiment #45 targeting locus DW showing profiles obtained with an external probe after NsiI digestion (WT: 10340bp; Rec: 11728bp).

Uncropped blots

Fig 4B

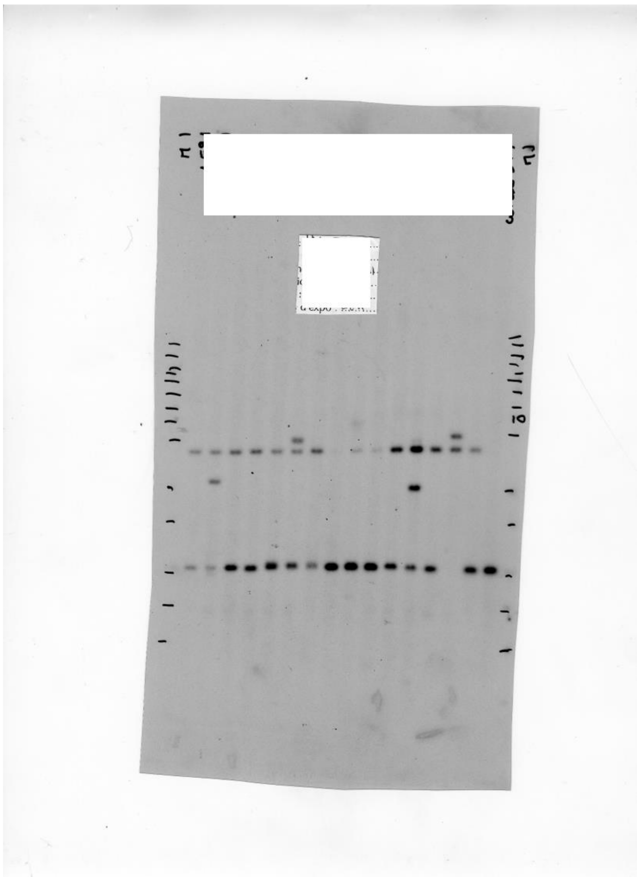

FigS4B

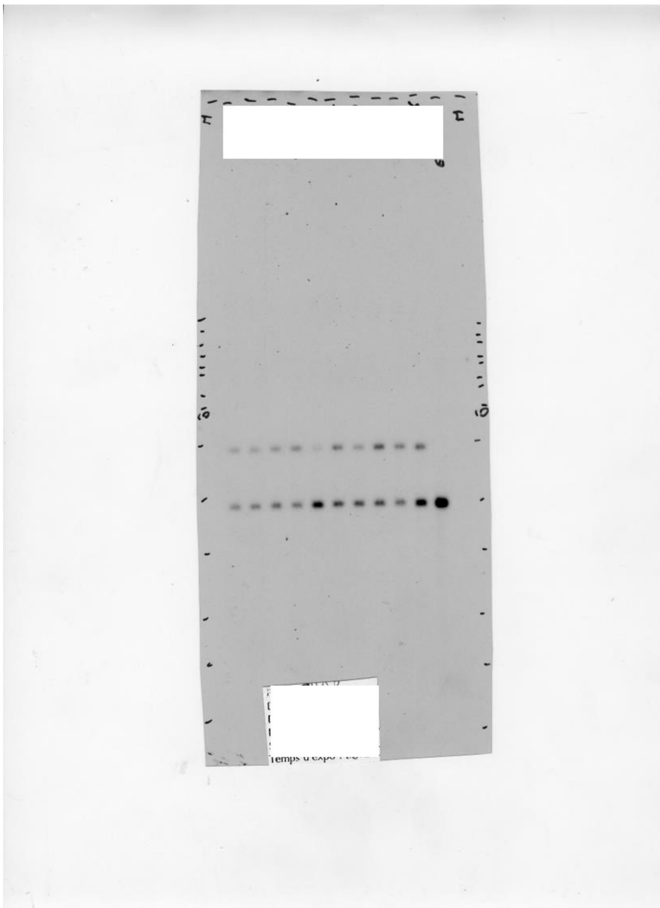

FigS4C

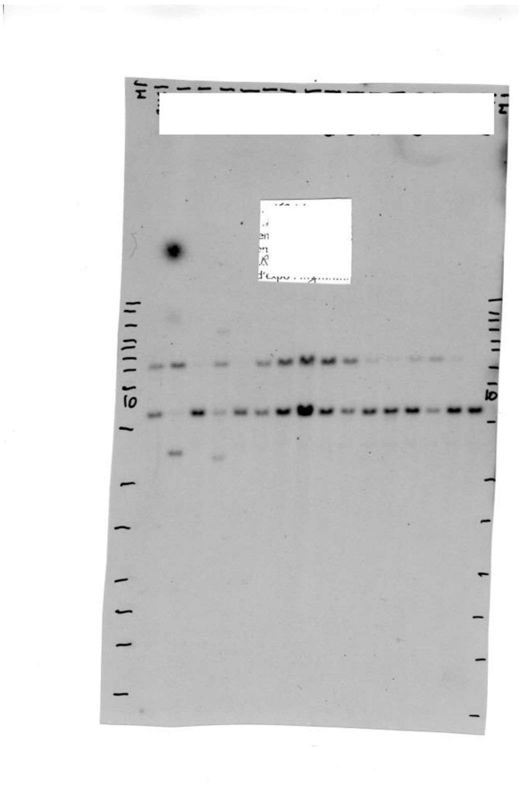

FigS4E

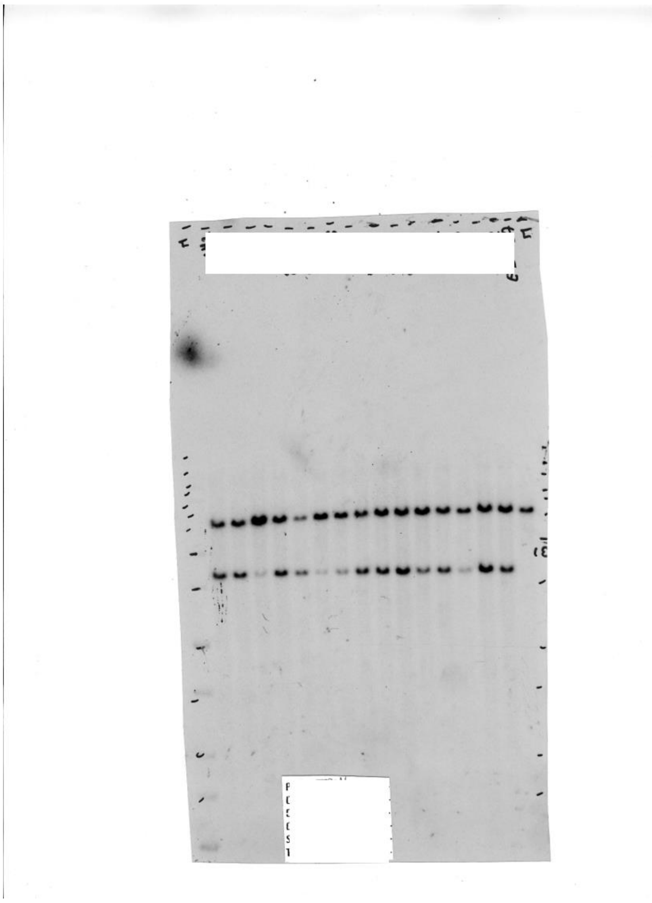

FigS4F

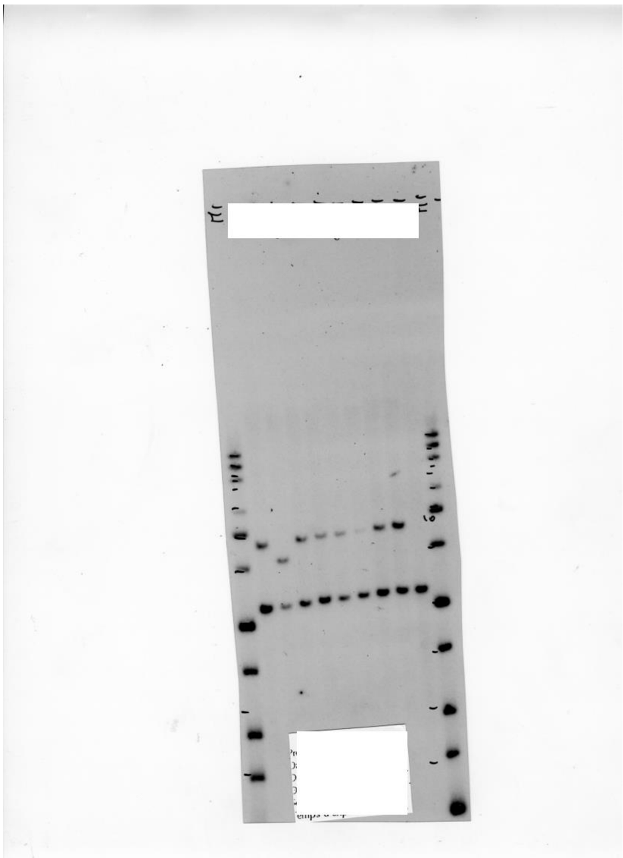

FigS4G

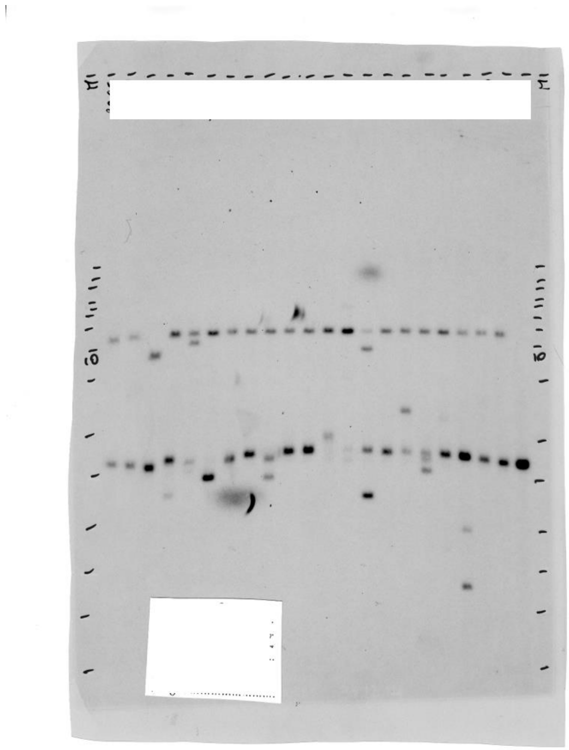

FigS4H

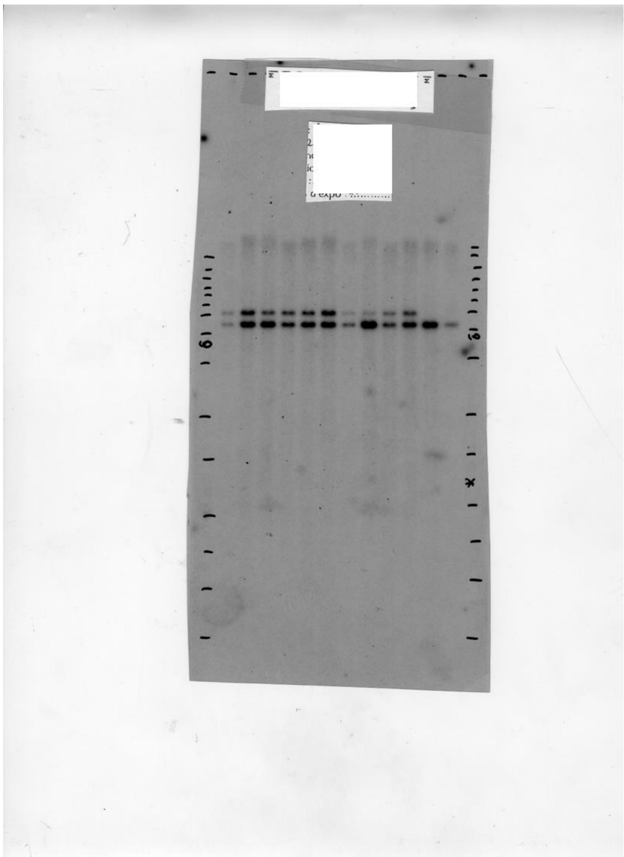

Supplement: Supplementary file 1 — Supplementary information [file 41598_2019_40181_MOESM1_ESM.pdf]
